# Ethical implications of digital communication for the patient-clinician relationship: analysis of interviews with clinicians and young adults with long term conditions (the LYNC study)

**DOI:** 10.1186/s12910-018-0250-0

**Published:** 2018-02-23

**Authors:** Agnieszka Ignatowicz, Anne-Marie Slowther, Patrick Elder, Carol Bryce, Kathryn Hamilton, Caroline Huxley, Vera Forjaz, Jackie Sturt, Frances Griffiths

**Affiliations:** 1 Warwick Medical School, The University of Warwick, Coventry, CV4 7AL UK; 2 Florence Nightingale Faculty of Nursing and Midwifery, King’s College London, London, UK

**Keywords:** Digital communication, Ethical issues, Young people

## Abstract

**Background:**

Digital communication between a patient and their clinician offers the potential for improved patient care, particularly for young people with long term conditions who are at risk of service disengagement. However, its use raises a number of ethical questions which have not been explored in empirical studies. The objective of this study was to examine, from the patient and clinician perspective, the ethical implications of the use of digital clinical communication in the context of young people living with long-term conditions.

**Methods:**

A total of 129 semi-structured interviews, 59 with young people and 70 with healthcare professionals, from 20 United Kingdom (UK)-based specialist clinics were conducted as part of the LYNC study. Transcripts from five sites (cancer, liver, renal, cystic fibrosis and mental health) were read by a core team to identify explicit and implicit ethical issues and develop descriptive ethical codes. Our subsequent thematic analysis was developed iteratively with reference to professional and ethical norms.

**Results:**

Clinician participants saw digital clinical communication as potentially increasing patient empowerment and autonomy; improving trust between patient and healthcare professional; and reducing harm because of rapid access to clinical advice. However, they also described ethical challenges, including: difficulty with defining and maintaining boundaries of confidentiality; uncertainty regarding the level of consent required; and blurring of the limits of a clinician’s duty of care when unlimited access is possible. Paradoxically, the use of digital clinical communication can create dependence rather than promote autonomy in some patients. Patient participants varied in their understanding of, and concern about, confidentiality in the context of digital communication. An overarching theme emerging from the data was a shifting of the boundaries of the patient-clinician relationship and the professional duty of care in the context of use of clinical digital communication.

**Conclusions:**

The ethical implications of clinical digital communication are complex and go beyond concerns about confidentiality and consent. Any development of this form of communication should consider its impact on the patient-clinician-relationship, and include appropriate safeguards to ensure that professional ethical obligations are adhered to.

## Background

There has been increasing support for the use of digital communication between patients and clinicians in the English National Health Care System (NHS). In 2012, the Department of Health made the introduction of secure electronic interaction between patients and their healthcare teams part of its official policy [1]. Several systematic and literature reviews have reported benefits of the use of digital communication in both adult and young populations [2–4], but the greatest potential benefit is arguably for adolescents with chronic health conditions who are at risk of disengagement with services [5]. However, the use of digital communication in these contexts raises a number of ethical concerns regarding confidentiality and consent, as well as potentially redefining the nature of the patient-clinician relationship. Confidentiality may become more difficult to enforce, as human curiosity continues to promote behaviour that derails even the most secure systems [6–8]. It may also be perceived as less important with the current prevalence of social media and the increasing trend to share personal information in public fora. The nature of the patient-clinician relationship as traditionally construed in a face-to-face encounter may need to be revised, with potential implications for the ethical and professional obligations that underpin this relationship [9, 10]. How is a professional duty of care, and its associated ethical obligations, to be interpreted in a digital world? Given these questions, and the growing importance of digital communication in health care delivery, there is limited evidence available about how digital communication influences the patient-clinician relationship and the corresponding ethical obligations. Published data indicate that digital communication can make this relationship closer and improve communication [2, 9, 11, 12], and even reduce the power differential between patient and clinician [13]. However, these methods of communication can also be viewed as impersonal [14–16]; the inherent lack of non-verbal communication can lead to misunderstanding [16]; or the patients may use asynchronous digital communication inappropriately for urgent issues requiring immediate clinical attention [14, 16] leading to potential serious harm. We do not know how digital clinical communication impacts on patient trust and autonomy or how it affects the perennial ethical tension for clinicians of balancing their responsibility to act in their patient’s best interests while respecting the patient’s autonomy [17, 18].

In 2003, The Council on Ethical and Judicial Affairs of the American Medical Association set out ethical guidelines for the use of electronic mail between patients and physicians [7]. The recommendations emphasised that the ethical obligations inherent in the clinician-patient relationship were the same whatever method of communication was used. Their focus was on providing appropriate information about email communication to patients, informed consent and maintaining confidentiality and privacy. Discussions on ethical aspects of digital communication between patients and their clinicians have continued to focus on these areas. However, there is little empirical evidence regarding patient and clinician experience of these technologies to inform this discussion, and to contribute to ethically informed development of the healthcare services that use these technologies. This paper identifies and explores, from the patient and clinician perspective, the ethical implications of the use of digital clinical communication in the context of young people living with long-term conditions. We use these results to contribute to the commentary on the ethical issues relating to the use of digital communication in these clinical contexts.

## Methods

We report on the ethical analysis undertaken within the LYNC study – a multi-site project exploring the effects, impacts, costs and necessary safeguards for young people with long-term conditions of engaging with NHS providers using digital communication in the clinical context [5]. The LYNC study was approved by the National Research Ethics Service Committee West Midlands – Black Country (Ref. 14/WM/0066). Written or verbal informed consent was obtained from all participants. Details of the study method have been reported elsewhere [5, 19]. It involved data collection from clinicians and patients across 20 NHS specialist clinics that provide care for young people (aged 16–24) with chronic physical or mental health conditions, such as cancer, sickle cell, liver disease, cystic fibrosis and psychosis. Interviews with participants were semi-structured and focussed on the participant’s experience of the use of digital communication between healthcare professional and patient in the context of management of the chronic condition. Interviews were transcribed, anonymised and uploaded into the NVivo programme [20] for coding and analysis. Interviewees were given the opportunity to contact the study team if they wished to read their interview transcripts.

Using an empirical ethics approach our analysis adopted an iterative process between the explicit and implicit ethical concerns expressed by participants and the ethical and professional norms that frame current conceptions of the patient-clinician relationship [21]. First, the team members read interview transcripts from two sites to identify examples of explicit articulation of ethical issues; areas of conflict or disagreement; expressions of discomfort with current or perceived practice; or examples of avoidance of an ethical issue. Implicit ethical issues or concerns were identified by the team drawing on ethical concepts such as autonomy, justice, and duty of care. This allowed us to identify an initial set of themes derived both inductively from the data and deductively using theoretical ethical concepts. These initial themes informed modification of the interview schedule to elicit reflection on the identified ethical issues. The modified interview schedule was used in a further three sites. We then coded all transcripts from these sites against the initial ethical themes identified with further discussion and refinement of the themes. The transcripts from these five sites were discussed in a series of analysis meetings with three members of the analysis team (AI, AS, PE). We then refined the themes in relation to ethical and professional normative frameworks. Overall, we analysed 59 interviews with young people and 70 with healthcare professionals. Finally, the process was repeated with a sample (around 10%) of transcripts from the remaining 15 LYNC study sites to look for any new themes/issues and to consolidate consensus on the initially agreed themes.

## Results

Clinicians and young people saw digital clinical communication as increasing patient empowerment and autonomy and reducing harm because of rapid access to clinical advice. However, some clinicians described difficulties with defining and maintaining boundaries of confidentiality, and blurring of the limits of their duty of care when unlimited access is possible. An overarching theme from the data was the need to re-conceptualise the patient-clinician relationship in the context of the use of digital communication. We discuss these findings in more detail below under three predominant themes: (1) autonomy and control, (2) defining the limits of duty of care, and (3) communication and trust (see Table 1).

**Table 1:** Key themes from the data

Key themes	Sub-themes discussed in the interviews
Autonomy and control	• Increasing young person’s control in the management of their condition. • Paradoxical reduced autonomy of young people and increased dependence on the clinician. • Opportunity to build a more personal relationship with their clinician more important than increased control. • Loss of clinician autonomy in relation to the timing and the style of the communication with young people; and control of information passing into the public domain.
Defining the limits of duty of care	• Ambiguity about when the duty of care is established and what is required by that duty. • Different views and ways of dealing with issues around duty of care amongst clinicians: • Establishing rules about access and responsiveness of clinicians to digital communication
Communication and trust	• DCC as an enabler of a trusting relationship between young people and their clinicians. • Importance to young people of face to face consultation for establishing trust. • Clinician concern regarding completeness of information provided by young people through digital communication. • Different understandings of confidentiality and privacy amongst young people and their clinicians.

### Autonomy and control

The majority of clinician participants agreed that digital clinical communication allowed young people to have more control in both the management of their condition and in the way in which they communicated with healthcare professionals. Easier access to expert advice gave them confidence in making decisions about their health, and increased options for communication enabled them to control the progress of difficult conversations. *‘They feel in control if they’ve got a question, they’re not disenfranchised. They’ve got someone to ask about it so they can feel empowered to ask those questions and control their own health rather than don’t know who to ask or whether to come forward with things.’* Cystic fibrosis 1 Specialist nurse 03

*‘…it gives people another chance to explain their issues and it can even be good for things that maybe you’re not comfortable talking about in front of the person…I think it is an invaluable resource because you know, you can ask anything, you don’t have to wait, you can get an answer.’* Liver Young person 10

Use of digital clinical communication appeared to bring about a shift in culture in the clinic with clinicians adopting methods of communication regularly used by young people rather than expecting young people to fit in with established health service determined methods of communication. They were adapting to the young person’s world rather than the young person being expected to adapt to their world.

*‘…if you had to rely on them ringing in the landline here, nine times out of ten it wouldn't happen because, you know, for a 14-year-old lad it's too much like hard work… I suppose it's bringing… not bringing yourself down but levelling… getting on the same level, the same wavelength.’* Mental health 3 (outreach team) Mental health practitioner 04

While some young people talked about achieving increased empowerment from digital clinical communication, in general they placed more emphasis on the increased opportunity to build a more personal relationship with their clinician through more frequent contact. Because of this the clinician was more likely to understand the young person’s circumstances and what was important to them.

*‘…it’s made [my relationship with the clinical team] stronger because I have more contact with them and more personal contact (…) they kind of know me a bit better and I know them a little bit better…So it doesn’t feel like I’m just another patient.’* Liver Young person 18

The responsiveness of digital communication made some young people feel more secure and cared for within the clinician-patient relationship.

*‘Well the fact that I could email instantly…being in contact with someone about something like that instantaneously eases your anxiety somewhat, just the fact that you know someone is going to read the email and provide some sort of response about what they think is going on. That’s sort of some reassurance.’* Sickle cell Young person 10

*‘[Young people] like to receive them because they know that somebody is caring about them and that it reminds them sometimes, oh actually yeah, there is something I want to ask you, I'm glad you've texted.’* Sickle cell Nurse specialist 12

Somewhat paradoxically, digital clinical communication could result in the reduced autonomy of young people. Some clinicians described young patients becoming more dependent rather than less dependent because of the increased access to clinical support and advice provided by digital communication. Easier access to advice via emails or text messages could remove the need for the young person to make decisions for themselves and thus disempower them from taking control of their own condition. This general dependence on easy access to a clinician could, in turn, develop into a more personal dependence on a specific clinician.

*‘I do think it is too…I think it makes patients a little too dependent on you and you only, which we don’t like to happen.’* Diabetes 1 Specialist nurse 03

*‘You know, they're not going to have me forever to text, so they need to learn to manage these situations themselves.*’ Mental health 3 (outreach team) Support worker 03

The use of digital communication raised some concerns about clinician autonomy for our clinician participants. Improved accessibility for patients can mean that a clinician has less control over both the timing and the style of the communication.

*‘…it allows you to be much more accessible, which is a great thing, but on the other hand it also prevents you putting in normal professional boundaries, that allow you to exist as a clinician actually.’* Mental health 1 (Early intervention) Consultant 09

*‘I've had text messages before where they just get a bit more friendly and they will sort of like put a kiss on the end and I just feel like it starts to get a bit more sort of friendly so it's a bit more harder to keep those boundaries in place with text messages.’* Mental health 4 (Early intervention) Assistant psychologist 15

Health care professionals also described concerns about losing control over the distribution of information when the content of the digital clinical communication was passed into the public domain by the patient without the consent of the clinician. This raised questions of whether the patient had a duty to respect the clinician’s privacy and the nature of the patient-clinician relationship with regard to sharing of and control over information.

*‘One young person decided to put texts that I'd been sending to her out on social media, which is inappropriate…And although she'd removed my name on all of the texts it was my…you know, they [other patients] knew it was what I'd said.’* Inflammatory bowel disease 2 Dietician 10

Our findings suggest that the impact of digital clinical communication on patient and clinician autonomy is complex. It has the potential to empower young people in managing their own care but it also provides them with an opportunity to avoid taking responsibility. It can challenge clinician autonomy in decisions about how and when information should be shared and with whom. One mechanism for facilitating both patient and clinician autonomy is to ensure that there is a clear process of informed consent prior to initiating this form of communication. Clinicians have an opportunity in this process to set out and negotiate the parameters of this method of communication, and patients have the information they need to make an informed choice about their participation in digital clinical communication. Some of our clinician participants had clear processes for obtaining consent but most were unaware of any institutional policies regarding use of digital clinical communication.

### Defining the limits of duty of care

Clinicians talked about facing difficult decisions when patients used text or email for communication relating to serious health concerns out of normal working hours:

*‘I got an email from (patient) in the middle of the night telling me she [the patient] was going to kill herself … That really raises questions of where does that put me responsibility-wise because that email was sitting there not accessed, and she’d actually given me that information, which if that was her calling in saying that to me, I’d be professionally obliged to do something with that.’* Liver Psychologist 01

This clinician was concerned that the duty of care was established with provision of information by the patient to the health professional, or by the fact that this form of communication had been agreed. This ambiguity about when the duty of care was established, and what is required by that duty, was shared by other clinician participants.

*‘You know, if you get an email, it would have to be acted on depending on what it said…so I think it raises a lot of issues like that about duty of care and about, you know, when are people checking them, how often do we check them, what will we do with the information.’* Mental health 2 (Child and adolescent mental health service). Psychologist 11

Some clinicians dealt with these concerns by having a strict rule about accessing their emails out of hours, arguing that the duty only materialised once they had received the information and not when it had been sent.

*‘If I’d chosen to read my work emails, as some people do at, you know, 10 o'clock on Saturday night, that I would then be professionally obliged to do something with that knowledge (…) It sounds awful but for my professional responsibility the worst case would have been if that event had happened, but also if I’d accessed it out of working times.’* Liver Psychologist 01

In the UK a legal duty of care is established when a therapeutic relationship is established between a clinician and a patient [22]. The General Medical Council requires that a doctor make the care of their patient their first concern, which would include responding to a clear expression of need by the patient [22]. However, for an appropriate response to occur there needs to be connection and communication with the patient. With more traditional forms of communication between patient and clinician (telephone and face-to-face encounter), the connection is immediate and confirmed, hence the duty of care established. With digital communication, there may be a misperception of established connection by the patient, and hence lack of clarity on whether a duty of care has been established. Our data show that clinicians using digital clinical communication differ in their views on when a duty of care is established.

Some clinicians using digital communication with their patients had very clear rules for themselves about accessing emails and texts out of working hours. These clinicians also emphasized the importance of making the limits of access and alternative sources of support clear to patients so that there was a joint understanding of the limits of the individual clinician’s duty of care.

*‘I will tell [patients] that you can contact me on all these things but you may not get an answer, because if you text and I'm in with somebody else for their one to one, I'm not going to be answering you until that's finished. ...so you know, we do accept those boundaries at the beginning.’* Mental health 3 (outreach team) Dialectical Behavioural Therapy co-ordinator 10

*‘We’ve put a kind of a note on the bottom of our emails explaining that we will pick up our emails during working hours Monday to Friday and that if there is anything urgent, that they need to go and see the GP or their local health provider.’* Liver Consultant 05

### Communication and trust

For some participants in our study, the use of digital communication enabled a trusting relationship to develop more easily.

*‘So she is, she's able…it enables her to be able to tell me how she's feeling and what she needs and how she needs it and her true feelings, where she can't do that when I'm sat in the room with her. So it's a useful tool for her.’* Mental health 3 (outreach team) Dialectical Behavioural Therapy co-ordinator 10

*‘I think it’s knowing that I can contact her easily and that she’s so nice about it all the time, she’ll always send a really friendly reply. I think I have a lot more trust in her, I feel quite confident in her care.’* Liver Young person 07

Not all patients were confident that digital communication increased trust. When the impact of decisions or communication of information was likely to have a significant effect on patient care a face-to-face encounter was seen as a better way of establishing the level of trust required.

*‘I’m trusting these people with my life; literally trusting these people with you know, with your life. This isn’t just a question of, you know, you’re asking somebody to do something for you, write a letter for you or something like that…So from a patient perspective I quite like to know who is treating me, who is calling the shots, and yes to be able to just discuss with them properly why they’re doing the things that they’re doing, or you know, what other options there are.’* Liver Young person 1

Clinicians also had concerns about communicating by digital means information that might have a major impact on the young person.

‘*You know, you could never text someone, oh by the way, you've progressed, because the way that it's dealt with is obviously incredibly important, especially the way that they take to it psychologically and that kind of thing. So I think for the more serious conversations, good or bad, they need to be done face to face.’* Mental health 4 (Early intervention) Case manager 03

Some participants, both young people and their clinicians, commented that it was not always possible to tell if the young person is withholding important information about their health in a text or email. Face to face or even telephone encounters provide less scope for pretending everything is well when it is not. This uncertainty created anxiety for clinicians and young people alike.

*‘…and I think also there are so many things that you could fabricate in an email or a text, you could say you feel fine when actually you’re not that fine…so I just don’t think you can replace that personal one-on-one.’* Liver Young person 16

*‘We have some people who say they'd prefer a text message. I don't feel happy about just sending a text which is why I will say, do you want a phone call…There's a couple of occasions where I've tried to press the point by saying, are you saying no because you don't want to speak or are you saying no because you feel fine? And then a couple of times I haven't had a response back, so that's even worse really because you think: well what does that mean, have they just turned their phone off.’* Mental health 3 (outreach team) Mental health practitioner 05

A key element of a trust relationship between patient and clinician is the expectation that the clinician will maintain confidentiality, and potential breaches of confidentiality are the main ethical concern expressed in the literature on digital clinical communication. Young people had different understandings of confidentiality and privacy, and expressed different levels of concern about possible breaches. Some told us that that they were worried about emails being intercepted or messages on the home screen of their phone seen by others.

*‘I don’t particularly publicise the fact that I have an illness…I mean texts flash up on people’s phones and people, you know, press the lock screen to see the time or whatever and you know, there’s a text from the hospital....’* Cancer 1 Young person 02

Others were less concerned, either trusting their doctor to limit communication to non-sensitive material, or placing less emphasis on the confidential nature of medical information compared to other personal information such as bank details.

*‘I mean, there’s nothing about, like, my bank details or things like that, it’s just, kind of, about my health. I’m not as worried about it.’* Kidney Young person 05

Clinicians were usually cautious about sending confidential data digitally because of concerns about data security. Some clinicians were less concerned about patients providing confidential information in this way, seeing this as the responsibility of the patient.

‘*If it's your data and you've made the choice to send it, then that's your look out, and I think that's why we're very careful about not sending anything via email. I suppose they've made the choice to send us the data by text and we would respond, but I wouldn't give any data out by text, I would only give it over the phone, which I feel is more secure.’* HIV Consultant 13

The expectation that patients will take responsibility for disclosing personal information in their communications requires that patients are aware of the risks. Several clinicians emphasised the importance of informing patients clearly about the implications of using digital communication and seeking their consent prior to commencing this service.

‘*I think there probably needs to be some sort of formal conversation or information about the use of email given to patients when they first attend for their first appointment, just to make sure that you’ve got their consent to communicate with email.’* Dermatology Consultant 02

However, most clinicians were unaware of any specific guidance on informing patients and obtaining consent for digital clinical communication.

Digital clinical communication can generate new potential risks of inadvertent breaches of confidentiality because of the ease of access of information. Health professionals who are vigilant about confidentiality in other contexts may not recognise these risks. Some clinicians described accessing email in public places such as on the bus or train:

*‘I tend to do emailing as I get on the train because my train’s a 40-minute train ride…I can finish off stuff that isn’t urgent.’* Liver nurse 04

This activity was identified as a risk by only one participant and only in relation to telephone calls.

Digital clinical communication can facilitate development of trust in the patient-clinician relationship by enabling the establishment of a more immediate and personal connection. However, the lack of direct personal contact between the clinician and patient could potentially weaken trust on the part of the clinician. This form of communication has challenges for data security and confidentiality which could threaten patient trust in their clinicians. Our data suggest that a trust relationship between patients and clinician can be nurtured by using digital clinical communication in addition to, rather than in place of face-to-face consultation, and being aware of, and honest about, risks to confidentiality.

## Discussion

Our findings suggests that the use of digital clinical communication has implications for three key elements of the patient-clinician relationship and the professional ethical framework that informs this relationship. It shifts the balance between patient and clinician autonomy, raises questions about the establishment and fulfilment of a duty of care, and creates new challenges regarding confidentiality.

Our current understanding of the patient-clinician relationship is one of shared decision making requiring values of openness and respect for patient autonomy and supporting patients to manage their own condition. Good communication is essential for this model of the patient-clinician relationship to be realised and opportunities to improve communication through use of digital technology have the potential to facilitate or improve this relationship. Previous studies have suggested that the use of digital clinical communication improves the patient-clinician relationship by enabling patients to feel more comfortable about disclosing information to their clinicians and by reducing the imbalance of power between patient and clinician [23]. Our data support this hypothesis but also suggest that there are other effects of digital communication on the patient-clinician relationship, and that the effect on patient autonomy is more complex than the current literature acknowledges.

Our empirical data do not suggest a conception of autonomy that is limited to individual patient control and empowerment, which is how autonomy is usually interpreted in healthcare. Digital clinical communication provided more choice for patients on how and when to access information and communicate with clinicians but this did not always mean they took more control over managing their illness. In addition, access to digital communication with their healthcare professionals can place additional responsibilities on patients, for maintaining confidentiality of shared communication, and for respecting boundaries of access. A more nuanced conception of autonomy may be more helpful when talking about the role of digital communication in the clinical context. For example, principled autonomy where autonomous choice is governed by principles of duties or obligations [24], recognises that patients have responsibilities both to themselves and others. This model of autonomy could require patients to respect professional boundaries. Another potentially useful model of autonomy in this context is relational autonomy which sees autonomy as embedded in social relationships (for example the patient-clinician relationship and respect for autonomy, not simply provision of opportunity to make choices) [25]. Our young people participants valued the deeper relationship with their clinician that digital clinical communication brought; a relationship that could provide a better understanding of how to support the young person to realise their autonomy. The digital communication is a first step in the process but in itself is not enough.

A key element of any relationship between a patient and their clinician is the clinician’s professional and legal duty of care to that patient. Our data show that clinicians using digital clinical communication experience substantial moral concern that they may not be able to fulfil their duty of care in the context of asynchronous communication. Other authors have identified this concern about limits of a duty of care with electronic communication. Recupero specifically considers this in psychiatric care but uses non-psychiatric legal cases to caution that a breach of duty of care could result from a delayed response to an email communication [26]. She and others stress the importance of having robust systems in place to provide access to clinical advice when patients contact clinicians by telephone or digital communication, and the necessity of providing clear information to patients about the limits and risks of using digital communication in a therapeutic relationship [26]. The need for guidelines on good practice in the use of digital communication, including informed consent and establishing safe systems for data confidentiality and patient care, is a recurrent theme in the literature on digital clinical communication. In our study many clinicians were unaware of any guidance either locally within their organisation, or nationally. In May 2016 NHS England issued guidance from their information governance team on the use of emails and text messaging for communicating with patients [27]. The guidance emphasises the need for explicit consent and provides a sample consent form. However, experience from the US suggests that guidance alone may not be sufficient for clinicians to change practice in adapting to the specific ethical challenges of digital clinical communication. A survey of physicians in the United States in 2003 found that of 204 who used email communication with patients at least once a day, 72% reported they never obtained informed consent for use of email [28] despite previously published guidance from the American Medical Association [14].

### Strengths and limitations of the study

A strength of our study is its inclusion of the perspectives of many young people and a wide range of clinicians with different experiences of the use of digital communication. The method used enabled us to explore specifically participants’ views on the ethical issues arising from the use of digital communication in some of our sites. However, while a large number of case study sites were included in the main study, the focus on ethical issues during data collection was limited to three sites. In our analysis we validated our themes against all other sites and our analysis resonated with the data from these sites, but this may still have limited the richness of the ethical reflection obtained. Overall our participants were enthusiastic users of digital clinical communication so our data may not have accurately captured the concerns of non-users of digital clinical communication.

## Conclusions

Digital technology is likely to become a key element in communication between patients and their clinicians in the future. It has the potential to facilitate patient autonomy and empowerment, and improve clinical care, but may result in a shift in the patient-clinician relationship that has implications for how we conceptualise the professional duty of care. As healthcare organisations and clinicians embrace these technologies for communicating with patients they will need to be clear to their patients, and to themselves, about the implications with regard to responsibilities for confidentiality, respecting boundaries, and strategies to ensure that patients are not harmed. Organisational guidelines should address these issues, and should ensure that patients are informed of any risks and limits to its use, and give consent. Education for health care professionals will be needed to accompany implementation of the guidelines so that they are aware of the ethical implications of the use of Digital clinical communication including their obligation to obtain informed consent from their patients. If our aim is to empower patients with regard to managing their health care we should also empower them to make informed choices about clinical communication.

## Abbreviations

LYNC study: The role of digital communication in patient-clinician communication for NHS providers of specialist clinical services for young people receiving specialist clinical services: a mixed methods study
NHS: National Health care System
NIHR: National Institute of Health Research
UK: United Kingdom
